# 基于生物信息学数据探究PKIG与肺鳞癌的相关性及其在肿瘤微环境中的作用

**DOI:** 10.3779/j.issn.1009-3419.2023.102.24

**Published:** 2023-07-20

**Authors:** Qing LIU, Haitian LI, Bin LI, Meiyu REN, Zhenqing LI, Yuzhen CHEN, Zhizhong ZHENG, Yuqi MENG, Haiming FENG

**Affiliations:** 730030 兰州，兰州大学第二医院胸外科，兰州大学第二临床医学院; Department of Thoracic Surgery, Lanzhou University Second Hospital, Lanzhou University Second Clinical Medical College, Lanzhou 730030, China

**Keywords:** 肺肿瘤, 生物信息学, 肿瘤微环境, 免疫治疗, cAMP依赖性蛋白激酶抑制剂γ, Lung neoplasms, Bioinformatics, Tumor microenvironment, Immunotherapy, cAMP-dependent protein kinase inhibitor γ

## Abstract

**背景与目的** 肺癌是全球癌症相关死亡的主要原因，患者从手术、放疗和化疗这些传统治疗方法中生存获益有限。免疫疗法作为肺癌的一种新兴治疗手段，显著延长了患者的总生存期（overall survival, OS），然而，仅有一部分患者能够从中获益，需要我们更深入地探索免疫治疗生物标志物以筛选优势人群。**方法** 从癌症基因组图谱（The Cancer Genome Atlas, TCGA）和基因表达综合数据库（Gene Expression Omnibus, GEO）下载原始数据，利用R软件和TIMER数据库筛选肺鳞癌（lung squamous cell carcinoma, LUSC）免疫预后相关基因。在TCGA和GEO数据库中研究了目标基因的表达情况，并通过R软件和TISIDB数据库对目标基因进行基因本体（Gene Ontology, GO）和京都基因与基因组百科全书（Kyoto Encyclopedia of Genes and Genomes, KEGG）富集分析以及与肿瘤免疫特征之间的相关性分析。**结果** 我们筛选出cAMP依赖性蛋白激酶抑制剂γ（protein kinase inhibitor gamma, PKIG）这个免疫预后相关基因，PKIG在LUSC和正常组织中的表达有明显差异，并对LUSC的诊断和预后评估具有重要参考价值。PKIG差异基因主要集中富集在体液免疫反应的调节等过程。PKIG的表达与调节性T细胞（regulatory T cells, Tregs）的浸润水平呈正相关（r=0.340, P<0.001）。此外，PKIG还与LUSC中趋化因子配体2（chemokine C-C motif ligand 2, CCL2）（r=0.503, P<0.001）、CXC趋化因子配体12（CXC chemokine ligand 12, CXCL12）（r=0.386, P<0.001）和CXC趋化因子受体4（CXC-chemokine receptor 4, CXCR4）（r=0.492, P<0.001）等趋化因子/趋化因子受体以及细胞程序性死亡蛋白1（programmed cell death protein 1, PDCD1）（r=0.359, P<0.001）、细胞毒性T淋巴细胞相关蛋白4（cytotoxic T-lymphocyte associated antigen 4, CTLA4）（r=0.375, P<0.001）和T细胞免疫球蛋白ITIM结构域（T cell immunoglobulin and ITIM domains, TIGIT）（r=0.305, P<0.001）等免疫抑制剂的表达水平呈正相关。**结论** 通过生物信息学分析筛选出LUSC免疫预后相关基因PKIG，PKIG与LUSC预后和免疫微环境高度相关，有望成为LUSC免疫治疗的潜在生物分子标志物。

## Abstract (en)

**Background and objective** Lung cancer is the leading cause of cancer-related death worldwide, and patients have limited survival benefits from traditional treatments such as surgery, radiotherapy and chemotherapy. As a new treatment for lung cancer, immunotherapy has significantly prolonged the overall survival (OS) of patients. However, only some patients can benefit from it. We need to explore immunotherapy biomarkers more deeply to screen for advantages. **Methods** The original data were downloaded from The Cancer Genome Atlas (TCGA) and Gene Expression Omnibus (GEO) database, and the immunological and prognostic genes of lung squamous cell carcinoma (LUSC) were screened using R software and TIMER database. The expression of target genes was studied in TCGA and GEO databases, and Gene Ontology (GO) and Kyoto Encyclopedia of Genes and Genomes (KEGG) enrichment analysis and correlation analysis with tumor immune characteristics were performed by R software and TISIDB database. **Results** We screened out a gene related to immunity and prognosis, cAMP dependent protein kinase inhibitor γ (PKIG), which is significantly differentially expressed in LUSC and normal tissues, and has important reference value for the diagnosis and prognosis assessment of LUSC. PKIG differential genes are mainly concentrated in the regulation of humoral immune response and other processes. The expression of PKIG was positively correlated with the infiltration level of regulatory T cells (Tregs) (r=0.340, P<0.001). In addition, the expression level of PKIG was positively correlated with the expression of chemokines/chemokine receptors such as chemokine C-C motif ligand 2 (CCL2) (r=0.503, P<0.001), CXC chemokine ligand 12 (CXCL12) (r=0.386, P<0.001) and CXC-chemokine receptor 4 (CXCR4) (r=0.492, P<0.001), and immunoinhibitors such as programmed cell death protein 1 (PDCD1) (r=0.359,P<0.001), cytotoxic T-lymphocyte associated antigen 4 (CTLA4) (r=0.375, P<0.001) and T cell immunoglobulin and ITIM domains (TIGIT) (r=0.305, P<0.001) in LUSC. **Conclusion** The immunological and prognostic gene PKIG in lung squamous cell carcinoma was screened through bioinformatics analysis. PKIG is highly correlated with LUSC prognosis and immune microenvironment, and is expected to be a potential biomolecular marker for LUSC immunotherapy.

肺癌是全球癌症相关死亡的主要原因^[1]^。肺癌分为两类：非小细胞肺癌（non-small cell lung cancer, NSCLC）和小细胞肺癌（small cell lung cancer, SCLC）。其中，NSCLC最常见，约占所有肺癌病例的85%^[2]^。手术、化疗、放疗和靶向治疗等传统治疗方法一直是NSCLC的标准治疗方法，然而，NSCLC患者从这些传统治疗方式中生存获益有限。免疫系统在抗肿瘤活性中起着至关重要的作用，在正常情况下，它可以识别癌细胞并在鉴定出肿瘤相关抗原后启动适当的反应以去除这些细胞，使癌症的治疗更接近精准医学^[3,4]^。多项临床研究^[5⇓-7]^结果表明，免疫疗法相比传统化疗具有更好的疗效和更少的副作用。然而，仅有一部分患者能够从免疫治疗中获益，更多潜在的免疫治疗生物标志物需要去探索以筛选优势人群。高通量测序技术作为一种新兴的肿瘤疾病检测手段，在基因组变异检测、临床应用等方面已经取得了显著的进展^[8]^。高通量测序技术有助于我们更好地理解肺鳞癌（lung squamous cell carcinoma, LUSC）的发病机理，并进一步筛选出用于LUSC诊断、治疗和预后评估的生物标志物。基于此，本研究采用生物信息学方法筛选出了癌症基因组图谱（The Cancer Genome Atlas, TCGA）和基因表达综合数据库（Gene Expression Omnibus, GEO）数据集中与LUSC预后和免疫相关的基因。此外，我们还探索了目标基因在LUSC组织中的表达情况以及与LUSC免疫微环境的关系，为LUSC免疫治疗提供了潜在的生物分子标志物。

## 1 资料与方法

### 1.1 数据收集

从GEO数据库（https://www.ncbi.nlm.nih.gov/geo/）中获取4个基因芯片（GSE33532、GSE30219、GSE21933、GSE3268）。GSE33532包含16个LUSC样本和4个正常肺组织样本。GSE30219包含61个LUSC样本和14个正常肺组织样本。GSE21933包含10个LUSC样本和20个正常肺组织样本。GSE3268包含5个LUSC样本和5个正常肺组织样本。从TCGA数据库（https://portal.gdc.cancer.gov）（36.0版）下载并整理TCGA-LUSC项目STAR流程的RNAseq数据以及临床数据，包括502个LUSC样本和49个正常肺组织样本。

### 1.2 免疫预后相关差异基因获取

使用R软件Limma包检测LUSC与正常组织之间的差异基因。FDR<0.05和|log2（FC）|>1.0认为差异有统计学意义，然后，利用在线韦恩图（https://jvenn.toulouse.inrae.fr/app/example.html）分析网站从TCGA、GSE33532和GSE30219这3个数据集中获得共表达差异基因。使用R survival包筛选出LUSC预后相关基因，将得到的预后相关基因导入TIMER数据库（https://cistrome.shinyapps.io/timer/）进一步筛选出免疫预后相关基因。

### 1.3 目标基因预后分析

使用R survival包分析确认cAMP依赖性蛋白激酶抑制剂γ（protein kinase inhibitor gamma, PKIG）表达和其他临床变量对总生存期（overall survival, OS）的影响，单因素中样本满足P<0.1就会进入到多因素Cox中构建模型。从Cox回归分析中选择独立的临床病理预后变量，使用R rms包构建Nomogram相关模型并进行可视化，以评估LUSC患者1、3和5年的OS率。受试者工作特征（receiver operating characteristic, ROC）曲线和时间依赖ROC使用R pROC包和timeROC包进行创建，结果用ggplot2包进行可视化。

### 1.4 目标基因功能富集分析

为探索PKIG可能参与的生物学过程和途径，我们将TCGA中PKIG表达水平的中位数作为截断值把LUSC样本分为高表达组和低表达组，使用DESeq2方法对两组测序数据进行差异分析，获得PKIG相关的差异表达基因。我们使用R clusterProfiler包对PKIG差异基因进行基因本体（Gene Ontology, GO）和京都基因与基因组百科全书（Kyoto Encyclopedia of Genes and Genomes, KEGG）以及基因集富集分析（Gene Set Enrichment Analysis, GSEA）。此外，从MSigDB数据库（https://www.gsea-msigdb.org/gsea/msigdb）下载了c2.cp.all.v2022.1.Hs.symbols.gmt子集合，用以评估相关途径和分子机制。结果P值使用Benjamini and Hochberg（BH）方法进行多重假设检验校正。所有分析结果都通过R ggplot2包进行可视化。

### 1.5 目标基因蛋白互作网络分析

STRING数据库（http://string-db.org）是一个基于公共数据库和文献信息的蛋白质相互作用网络数据库，Cytoscape（v3.9.1）是一个用于可视化分子相互作用网络和生物途径，并将这些网络与注释、基因表达谱和其他状态数据集成在一起的软件。使用STRING数据库和Cytoscape软件构建PKIG与其相关基因之间的蛋白质互作网络（protein-protein interaction networks, PPI）。

### 1.6 目标基因免疫浸润分析

通过单样本基因集富集分析（single-sample gene set enrichment analysis, ssGSEA）算法对LUSC样本进行免疫浸润分析，使用GSVA包对24种免疫细胞进行分析。通过Spearman相关系数确定PKIG和每个免疫细胞亚群之间的关系。

### 1.7 目标基因TISIDB数据库分析

TISIDB数据库（http://cis.hku.hk/TISIDB/）是一个通过整合研究文章和多种类型的高通量数据建立的关于肿瘤-免疫相互作用的数据库。我们使用TISIDB数据库的“免疫调节剂”模块和“趋化因子”模块分析了PKIG表达水平与免疫检查点基因和趋化因子/趋化因子受体表达水平之间的相关性。

### 1.8 统计学分析

箱式图用于评估LUSC患者中PKIG基因的表达水平，数据表示为中位数和四分位数间距（interquartile range, IQR）。通过Wilcoxon秩和检验分析两组之间的差异。使用R survival包和survminer包生成Kaplan-Meier生存曲线，并使用对数秩检验进行检测。此外，还计算了95%置信区间的风险比（hazard ratio, HR）和对数秩P值。通过单因素Cox回归分析评估可能的预后因素，并利用多因素Cox回归分析确认PKIG表达与其他临床变量共同对生存率的影响。统计分析均使用R软件（4.2.1版）。P<0.05认为差异有统计学意义。

## 2 结果

### 2.1 LUSC中差异基因的鉴定

在GSE33532中，共筛选出3156个差异基因，包括1462个上调基因和1694个下调基因。GSE30219中筛选出2410个差异基因，其中1057个上调基因和1353个下调基因。在TCGA中，共鉴定出16,058个差异基因，其中10,565个上调基因和5493个下调基因。然后，利用维恩图网站分析这3个数据集的差异基因，最后筛选出1465个共表达差异基因，包括688个上调基因和777个下调基因（图1）。

**图1:**
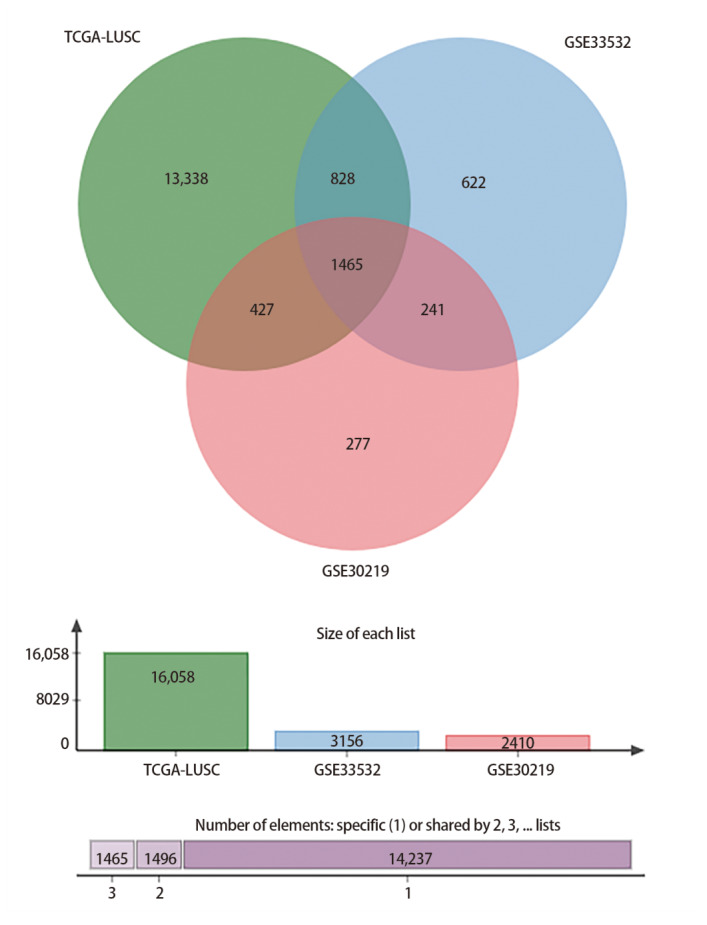
TCGA和GEO数据集共表达差异基因

### 2.2 免疫预后相关差异基因筛选

根据基因表达水平的中位数将LUSC样本分为高表达组（前50%样本）和低表达组（剩余50%样本）。使用R语言对共表达差异基因进行预后分析并生成Kaplan-Meier生存曲线图，共得到157个预后相关基因[PKIG、纤维胶凝蛋白3（ficolin 3, FCN3）、Wolfram综合征蛋白1（Wolfram syndrome 1, WFS1）、中间α-球蛋白抑制因子H3（inter-alpha-trypsin inhibitor heavy chain 3, ITIH3）和桥粒胶蛋白3（desmocollin 3, DSC3）等]（图2）。在TIMER数据库中分析这些基因与6种免疫细胞的相关性，结果显示，PKIG、锌指E盒结合同源盒蛋白2（zinc finger E-box binding homeobox 2, ZEB2）、CD93分子（CD93 molecule, CD93）、含铜胺氧化酶3（amine oxidase, copper containing 3, AOC3）、巨噬细胞清道夫受体1（macrophage scavenger receptor 1, MSR1）、血小板/内皮细胞黏附分子1（platelet/endothelial cell adhesion molecule 1, PECAM1）和激活素受体样激酶1（activin A receptor type II-like 1, ACVRL1）等29个基因在LUSC中与免疫相关（图3）。其中，PKIG基因在LUSC肿瘤微环境（tumor microenvironment, TME）中的作用鲜见报道。

**图2:**
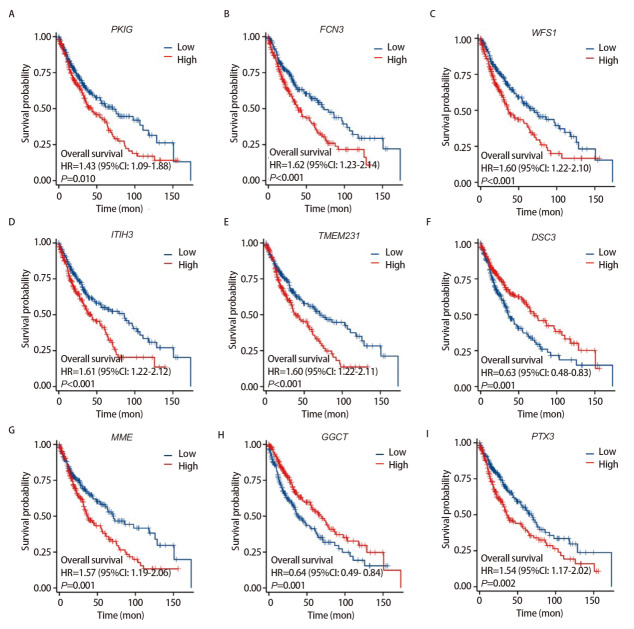
LUSC中PKIG（A）、FCN3（B）、WFS1（C）、ITIH3（D）、TMEM231（E）、DSC3（F）、MME（G）、GGCT（H）和PTX3（I）的Kaplan-Meier生存分析。

**图3:**
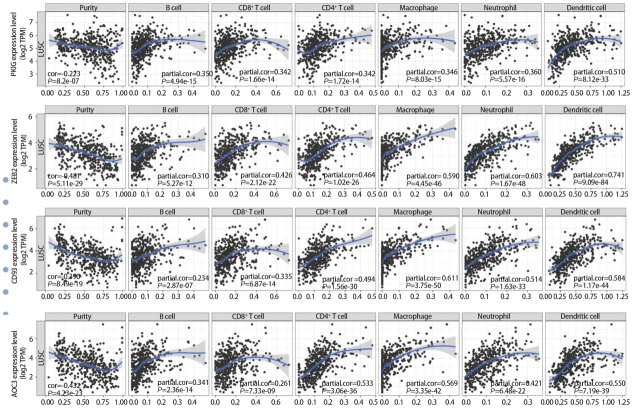
免疫相关基因与TIMER数据库中免疫细胞的相关性分析

### 2.3 PKIG的差异表达

为了探索PKIG在正常组织和肿瘤中的表达水平，我们使用R软件包从TCGA下载并分析了PKIG在不同肿瘤和正常组织中的表达水平。结果表明，PKIG在肾上腺皮质癌、肝细胞肝癌、胰腺癌和黑色素瘤等的表达水平高于正常组织。此外，在食管癌、肺腺癌、肺鳞癌和结肠癌等肿瘤中观察到较低的表达水平（图4A）。我们将PKIG在LUSC与癌旁组织中的表达水平进行比较，结果显示，PKIG在LUSC中的表达水平显著低于癌旁组织（P<0.001；图4B）。其结果在LUSC和配对癌旁组织中也得到验证（P<0.001；图4C）。此外，我们还分析了LUSC患者中PKIG的表达与OS之间的相关性，分析显示，PKIG的表达水平与OS（P<0.05；图4D）显著相关。

**图4:**
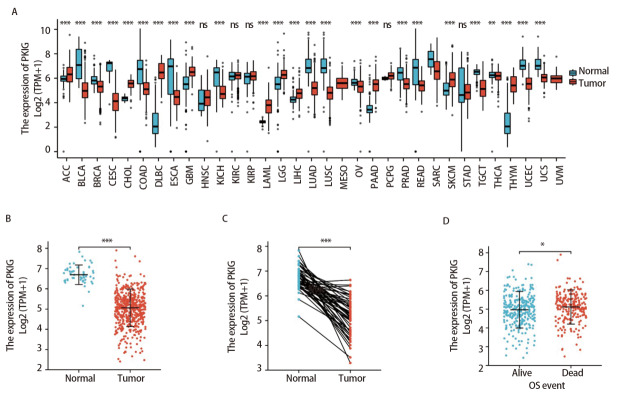
PKIG的差异表达。A：TCGA数据库中不同癌症数据集中的PKIG表达与正常组织对比；B：PKIG在TCGA数据库非配对样本中的差异表达；C：PKIG在TCGA数据库配对样本中的差异表达；D：TCGA数据库中LUSC患者的PKIG表达水平与OS的相关性。ns：无统计学差异；*：P<0.05；**：P<0.01；***：P<0.001。

### 2.4 使用独立的外部数据集进行验证

为了进一步验证PKIG在LUSC中的表达水平，我们选择了另外两个独立的外部GEO数据集（验证队列）来分析LUSC中癌症组织和癌旁组织的PKIG表达水平，包括GSE21933和GSE3268。结果显示，在非配对样本中LUSC的PKIG表达水平显著低于癌旁组织（GSE21933，P<0.001；图5A）。其结果在LUSC和配对癌旁组织中也得到验证（GSE3268，P<0.05；图5B）。这些数据验证了PKIG在LUSC中的低表达。

**图5:**
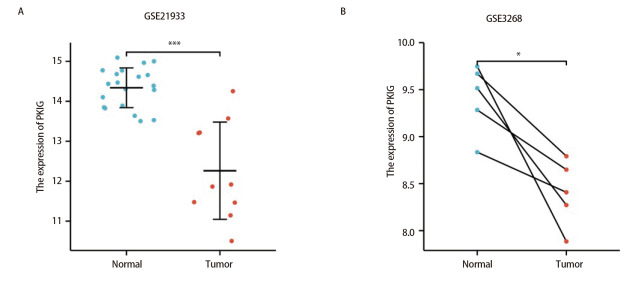
外部数据集验证。A：PKIG在非配对样本验证集中的差异表达；B：PKIG在配对样本验证集中的差异表达。*：P<0.05；***：P<0.001。

### 2.5 PKIG是LUSC的一个独立预后指标

我们通过Cox回归分析探究了PKIG表达和其他临床变量对OS的影响。单因素Cox模型显示，高PKIG表达、T分期、M分期和病理分期以及疾病治疗结果稳定和进展与较差的OS密切相关（P<0.05；表1）。进一步将这些因素进行多变量Cox回归分析，分析结果显示，PKIG表达、T分期和主要治疗结果与OS显著相关（P<0.05；表1）。以上数据表明，PKIG表达可以作为与LUSC患者OS相关的一个独立预后因素。

**表1:** 临床特征的单变量和多变量Cox回归分析

Characteristics	n	Univariate analysis			Multivariate analysis	
		HR (95%CI)	P		HR (95%CI)	P
Gender	496					
Female	130	Reference				
Male	366	1.211 (0.879-1.669)	0.241			
Age (yr)	490					
≤65	190	Reference				
>65	300	1.279 (0.960-1.704)	0.093		1.132 (0.722-1.776)	0.588
Smoker	484					
No	18	Reference				
Yes	466	0.585 (0.259-1.325)	0.199			
T stage	496					
T1+T2	403	Reference				
T3+T4	93	1.658 (1.200-2.291)	0.002		2.277 (1.278-4.059)	0.005
N stage	490					
N0	317	Reference				
N1+N2+N3	173	1.151 (0.869-1.523)	0.327			
M stage	415					
M0	408	Reference				
M1	7	3.112 (1.272-7.616)	0.013		2.100 (0.689-6.406)	0.192
Pathologic stage	492					
I+II	402	Reference				
III+IV	90	1.570 (1.139-2.163)	0.006		0.880 (0.455-1.703)	0.705
Primary therapy outcome	357					
PD+SD	47	Reference				
PR+CR	310	0.281 (0.186-0.422)	<0.001		0.150 (0.077-0.295)	<0.001
Residual tumor	411					
R0	395	Reference				
R1+R2	16	2.014 (0.885-4.583)	0.095		1.254 (0.302-5.208)	0.756
Anatomic neoplasm subdivision	236					
Central	144	Reference				
Peripheral	92	1.240 (0.829-1.855)	0.294			
PKIG	496					
Low	247	Reference				
High	249	1.400 (1.066-1.838)	0.015		2.019 (1.292-3.156)	0.002

TNM: Tumor-node-metastasis; PD: progressive disease; SD: stable disease; PR: partial response; CR: complete response; R0: complete tumor resection; R1: residual tumor was observed microscopically; R2: residual tumor was observed visually.

### 2.6 PKIG表达在LUSC中的诊断价值

为了分析PKIG表达在LUSC中的诊断价值，我们绘制了ROC曲线，并对TCGA数据库中的PKIG基因表达数据进行了列线图分析。ROC曲线是以假阳性率为横坐标，真阳性率为纵坐标绘制出来的曲线。ROC曲线下面积（area under the curve, AUC）常用于诊断试验的评估，AUC越接近于1，说明该变量在预测结局上诊断效果越好。在本研究中，PKIG的AUC值为0.968，表明变量PKIG的预测能力有较高准确性，如图6A所示。此外，我们还创建了变量PKIG的时间依赖ROC曲线，以预测1、3和5年的生存率。所有这些AUC值均>0.50，变量PKIG被认为适合预测（图6B）。此外，预后列线图结果表明，PKIG表达水平的预测能力优于T分期、N分期、病理分期、性别和年龄这些传统临床特征（图6C）。

**图6:**
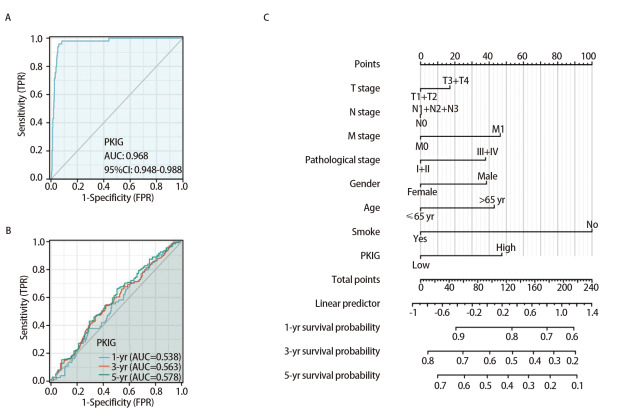
PKIG在LUSC中的诊断价值。A：PKIG表达的ROC分析；B：PKIG表达的时间依赖ROC分析；C：预后列线图。

### 2.7 LUSC中PKIG差异基因功能富集分析

为了了解PKIG在LUSC中的生物学功能，我们对PKIG差异基因中满足|log2(FC)|>1.5且P.adj<0.05阈值的781个基因进行GO和KEGG富集分析。分析发现这些基因集中富集在体液免疫反应的调节、免疫球蛋白的构成、受体配体活性的调控和神经活性受体-配体相互作用等过程（图7A，图7B）。此外，我们还将PKIG差异基因进行了GSEA分析，结果显示PKIG的高表达与多种通路相关，包括CD22介导的B细胞受体调控、FCGR3A介导的白介素（interleukin, IL）-10合成、补体的初始触发、B细胞受体的信号转导、Fceri介导的丝裂原活化蛋白激酶（mitogen-activated protein kinase, MAPK）激活和清道夫受体对配体的结合与摄取等（图7C-图7H）。

**图7:**
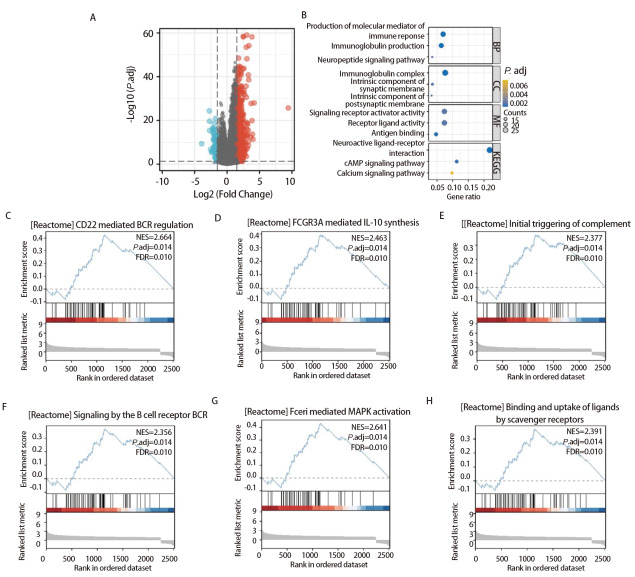
PKIG差异基因功能富集分析。A：PKIG差异基因火山图；B：PKIG差异基因GO和KEGG富集分析气泡图；C-H：PKIG差异基因GSEA分析折线图。

### 2.8 PKIG及其相关基因PPI分析

我们利用STRING网站和Cytoscape软件绘制了PKIG与其相关基因之间的PPI网络。PPI分析结果表明，PKIG与cAMP依赖性蛋白激酶抑制剂α（protein kinase inhibitor alpha, PKIA）、cAMP依赖性蛋白激酶抑制剂β（protein kinase inhibitor beta, PKIB）、腺苷脱氨酶（adenosine deaminase, ADA）和尿苷胞苷激酶1（uridine-cytidine kinase 1, UCK1）等10个不同基因存在蛋白互作关系，这些基因多数和cAMP信号通路有关（图8）。

**图8:**
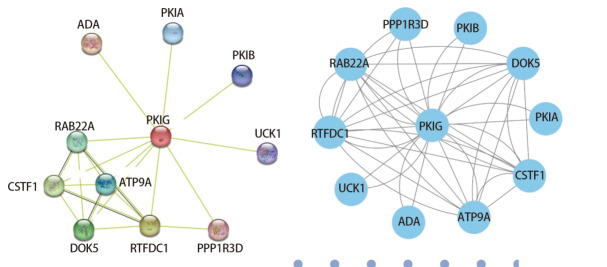
蛋白质-蛋白质相互作用网络分析

### 2.9 PKIG表达与免疫特性的相关性

我们使用ssGSEA算法分析了PKIG的表达水平与LUSC免疫浸润之间的相关性，结果显示PKIG表达水平与Tregs（r=0.340, P<0.001）、巨噬细胞（r=0.472, P<0.001）、自然杀伤细胞（r=0.511, P<0.001）和Th1辅助细胞（r=0.451, P<0.001）等免疫细胞的浸润水平呈正相关（图9）。

**图9:**
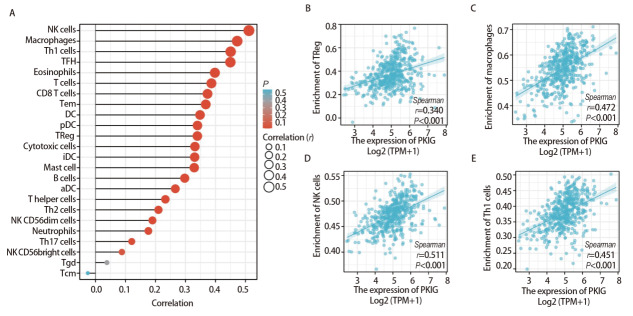
24种免疫细胞的相对丰度与PKIG表达水平之间的相关性。A：相关性棒棒图；B-E：Tregs、巨噬细胞、自然杀伤细胞和Th1辅助细胞浸润水平与PKIG表达之间的相关性散点图。

此外，我们使用TISIDB数据库分析了LUSC中PKIG的表达水平与免疫细胞趋化因子/趋化因子受体之间的相关性。热图结果显示，LUSC中PKIG的表达水平与CCL2（r=0.503, P<0.001）、CCL3（r=0.378, P<0.001）、CCL5（r=0.358, P<0.001）、CXCL12（r=0.386, P<0.001）、CCR1（r=0.402, P<0.001）、CCR2（r=0.449, P<0.001）、CXCR3（r=0.384, P<0.001）和CXCR4（r=0.492, P<0.001）等多种趋化因子和趋化因子受体的表达明显正相关。随后，我们使用TISIDB数据库分析了人类不同类型癌症中PKIG表达水平与免疫调节剂表达之间的相关性。结果提示，PKIG的表达与细胞程序性死亡蛋白1（programmed cell death protein 1, PDCD1）（r=0.359, P<0.001）、细胞毒性T淋巴细胞相关蛋白4（cytotoxic T-lymphocyte associated antigen 4, CTLA4）（r=0.375, P<0.001）、T细胞免疫球蛋白ITIM结构域（T cell immunoglobulin and ITIM domains, TIGITs）（r=0.305, P<0.001）和甲型肝炎病毒细胞受体2（hepatitis A virus cellular receptor 2, HAVCR2）（r=0.463, P<0.001）等免疫抑制剂的表达水平呈正相关（图10，图11）。这些结果提示，PKIG的高表达可能与癌症的发展有关。

**图10:**
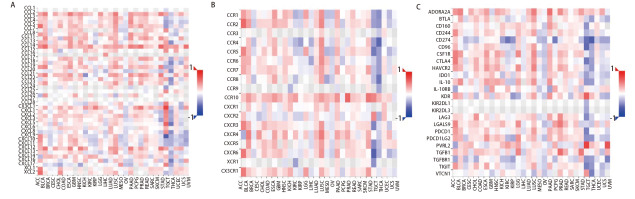
肿瘤中PKIG与趋化因子（A）、趋化因子受体（B）和免疫抑制剂（C）的相关性热图分析

**图11:**
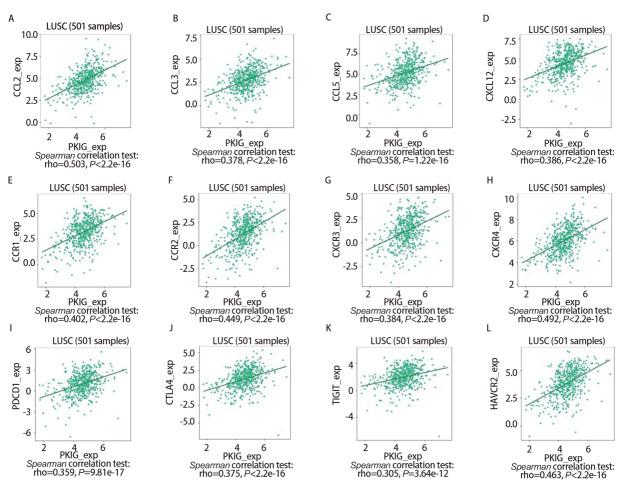
LUSC中PKIG与CCL2（A）、CCL3（B）、CCL5（C）、CXCL12（D）、CCR1（E）、CCR2（F）、CXCR3（G）、CXCR4（H）、PDCD1（I）、CTLA4（J）、TIGIT（K）和HAVCR2（L）的表达呈正相关。

我们从相关文献中下载了三级淋巴结构（tertiary lymphoid structures, TLSs）的39个标记基因，对PKIG基因和这39个标记基因进行相关性分析^[9]^。结果表明PKIG表达水平与可诱导共刺激分子（inducible T-cell co stimulator, ICOS）（r=0.473, P<0.001）、独立生长因子1（growth factor independence 1, GFI1）（r=0.424, P<0.001）、CD5（r=0.451, P<0.001）和CCR5（r=0.426, P<0.001）等基因的表达呈明显正相关（图12A-图12E）。PKIG高表达患者CCL8、CCL19、CXCL9、CD200、FBLN7、ICOS、GFI1和白介素1受体1（interleukin 1 receptor 1, IL-1R1）等标记基因的表达水平明显高于PKIG低表达患者（图12F）。以上结果表明，PKIG可能与TLS的生成高度相关。

**图12:**
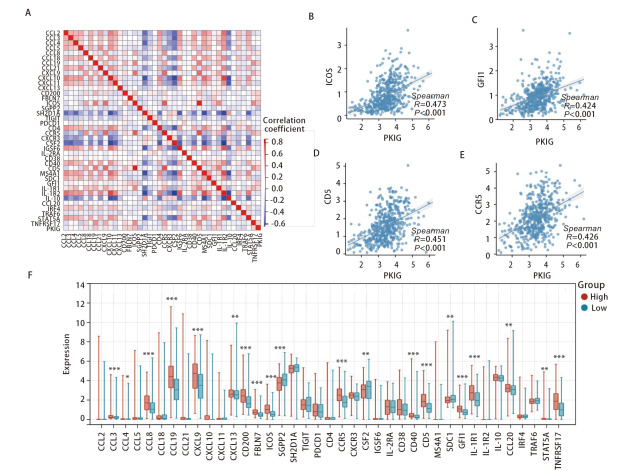
LUSC中PKIG与TLS标记基因的相关性分析。A：相关性热图；B-E：LUSC中PKIG与ICOS、GFI1、CD5和CCR5的表达呈正相关；F：PKIG高低表达组之间TLS标记基因的表达差异。*：P<0.05；**：P<0.01；***：P<0.001。

## 3 讨论

本研究通过生物信息学分析，鉴定出LUSC免疫预后相关基因PKIG。PKIG在蛋白激酶活性的负性调节、RNA聚合酶II转录的负性调节以及磷酸化等生物学过程中发挥着重要作用。有报道^[10]^显示，PKIG过表达能够增加他莫昔芬耐药乳腺癌细胞中ErbB7的表达水平。此外，还有研究^[11]^表明，PKIG与U87胶质瘤细胞中葡萄糖剥夺过程的响应有关。然而，目前还没有PKIG在肺癌中的相关报道。

在本研究中，我们通过整合多种生物信息学分析方法确定了PKIG在LUSC中的生物功能和潜在的调控途径。我们首先确定了PKIG基因在癌症中的表达和预后价值，并发现PKIG的表达水平在LUSC中显著下调。在LUSC中，PKIG表达水平与OS显著相关，并且是LUSC患者OS的一个独立预后因素。为了探索PKIG对LUSC预后的潜在调控途径，我们对PKIG差异基因进行了GO和KEGG以及GSEA富集分析。结果显示，这些基因集中富集在体液免疫反应的调节、免疫球蛋白的构成、受体-配体活性的调控和神经活性受体-配体相互作用等过程。并且，PKIG的高表达还与CD22介导的B细胞受体调控、FCGR3A介导的IL-10合成、补体的初始触发和B细胞受体的信号转导等免疫通路密切相关。这提示，PKIG可能与LUSC的免疫微环境相关，并且可能通过这些免疫过程参与调节LUSC的进展和抗肿瘤免疫反应的效率。

以往的研究^[12⇓-14]^表明，癌症的发生、发展与TME密切相关。其中，免疫细胞占TME的很大一部分，因此它们在介导促肿瘤和抗肿瘤免疫反应中起着关键作用。我们的研究发现，PKIG的表达与Tregs浸润水平呈正相关。在肺癌中，Tregs表达高亲和力IL-2受体与IL-2结合并螯合IL-4，以降低其对效应T细胞的作用。此外，Tregs还可以通过产生免疫抑制细胞因子、颗粒酶和穿孔素来抑制免疫力^[15⇓-17]^。同时，Tregs在肿瘤、转移性淋巴结和外周血等多个组织中的数量增加，活性增强，这与NSCLC的分期以及转移和复发的发生高度相关^[18,19]^。由此看来，PKIG可能在TME重塑以及LUSC的发生和转移过程中发挥重要作用，并且与LUSC的不良预后有关。

趋化因子是负责免疫细胞运输和淋巴组织发育的细胞因子亚家族，通过塑造肿瘤免疫和生物表型，而影响肿瘤的进展、治疗和患者预后^[20,21]^。在本研究中，我们发现，PKIG的表达水平与CCL2、CCL5、CXCL12、CCR2和CXCR4等趋化因子/趋化因子受体呈明显正相关。CCL2、CCL3和CCL5可以促进癌细胞的增殖、运动和上皮间质转化。此外，这些趋化因子还可将髓源性抑制细胞（myeloid-derived suppressor cells, MDSCs）和肿瘤相关巨噬细胞募集到TME中，进而促进和维持人类肿瘤干性^[22⇓-24]^。CXCL12-CXCR4信号通路在肿瘤增殖、转移和干性中发挥重要作用。CXCL12通过靶向血管内皮细胞，与血管内皮生长因子协同作用，促进肿瘤血管生成。同时，CXCL12-CXCR4趋化因子轴还有助于单核细胞MDSCs的募集^[24]^。在TME中，MDSCs能够抑制T细胞和NK细胞，并通过释放CCL3、CCL4和CCL5促进Tregs的募集。这可能解释了PKIG调节LUSC免疫浸润的机制。此外，PKIG还与LUSC中PDCD1、CTLA4、TIGIT和HAVCR2等免疫抑制剂的表达水平呈正相关。LUSC中PKIG与这些免疫负性调节因子的显著正相关性，提示了PKIG的高表达可能与癌症的发展有关，且该基因有可能并不是单独起作用，而是被动调控的。因此，虽然PKIG在LUSC中相对于正常组织低表达，但是在肿瘤患者中PKIG低表达者相比于高表达者预后更好。

最近的一些研究^[25⇓⇓-28]^发现，TLS对免疫检查点抑制剂（immune checkpoint inhibitors, ICIs）的反应提供了预测价值。TLS，是出生后在非淋巴组织中出现的免疫细胞的组织聚集体。黑色素瘤、肾细胞癌、软组织肉瘤和尿路上皮癌的治疗前活检中存在TLS和活跃的B细胞浸润已被证明与PDCD1抑制剂或PDCD1抑制剂联合CTLA4治疗的阻断反应相关。在探索PDCD1抑制剂新辅助治疗NSCLC的研究^[29]^中，研究者们观察到在消退性病变中TLS大量增加。本研究中，我们分析发现，PKIG的表达与多个TLS标记基因呈明显正相关。基于此，我们推测，PKIG可能和TLS的生成有关，PKIG高表达患者更有可能对ICIs治疗产生响应。

然而，尽管我们对PKIG进行了系统地分析，并使用不同的数据库进行了交叉验证，但我们仍然缺乏直接证据表明PKIG通过参与免疫浸润影响患者预后。我们将在未来的实验中进一步验证PKIG基因的功能。

总之，我们的研究发现PKIG的表达水平在LUSC中显著下调，并对LUSC的诊断和预后具有一定的参考价值。PKIG可能通过重塑TME影响LUSC的进展，有望成为LUSC患者免疫治疗的潜在生物分子标志物。

**Competing interests**

The authors declare that they have no competing interests.

**Author contributions**

Li B, Liu Q and Li HT conceived and designed the study. Liu Q, Li HT and Ren MY performed the experiments. Liu Q, Li ZQ and Chen YZ analyzed the data. Ren MY, Chen YZ and Zheng ZZ contributed analysis tools. Li ZQ, Meng YQ and Feng HM provided critical inputs on design, analysis and interpretation of the study. All the authors had access to the data. All authors read and approved the final manuscript as submitted.
